# Autoimmune thyroid disease disrupts immune homeostasis in the endometrium of unexplained infertility women—a single-cell RNA transcriptome study during the implantation window

**DOI:** 10.3389/fendo.2023.1185147

**Published:** 2023-07-12

**Authors:** Jilai Xie, Aiyuan Gu, Huangyi He, Qiaohang Zhao, Ya Yu, Jian Chen, Zhangliang Cheng, Ping Zhou, Qi Zhou, Min Jin

**Affiliations:** ^1^ Second Affiliated Hospital, School of Medicine, Zhejiang University, Department of Reproductive Medicine, Hangzhou, China; ^2^ Ministry of Education (MOE) Laboratory of Biosystems Homeostasis & Protection and Zhejiang Provincial Key Laboratory for Cancer Molecular Cell Biology, Life Sciences Institute, Zhejiang University, Hangzhou, China; ^3^ Department of Neuroscience and Developmental Biology, University of Vienna, Vienna, Austria

**Keywords:** autoimmune thyroid disease, unexplained infertility, endometrial receptivity, window of implantation, single-cell RNA sequencing

## Abstract

**Objective:**

Autoimmune thyroid disease (AITD) is known to be associated with unexplained infertility in women. Although the presence of antithyroid antibodies have been speculated to be a marker of an immune imbalance that might lead to implantation failure, its underlying mechanism influencing the endometrial receptivity remains to be elucidated. In this study, we used single-cell RNA sequencing (scRNA-seq) to dissect immune microenvironment in endometrium of AITD patients during window of implantation (WOI).

**Methods:**

We collected CD45^+^ immune cell populations of endometrium samples of unexplained infertile women with AITD (*n*=3), as well as samples of AITD^-^ controls (*n*=3). The cells were then processed with 10X Genomics Chromium for further analysis.

**Results:**

We characterized 28 distinct immune cell subtypes totally, and uncovered differences in the composition and gene expression patterns between AITD patients and controls. The proportions of T CD4^+^, cNK, ILC3, T CD8^+^ *GZMK^+^*, T CD8^+^ Cytotoxic and ILC3 *CD3E* ^-^ cells were increased, and CD366^+^ uNK1 was decreased in AITD^+^ patients. And the abnormal expression of *GNLY* and chemokines was observed in AITD patients. In addition, uNK and T CD8^+^ Cytotoxic cells showed lower cytotoxicity but activation of immune response. Genes enriched in cell adhesion of ILC3 and Tregs were downregulated, while the number of ILC3 and Tregs were increased.

**Conclusion:**

Immune imbalance exists in endometrium during WOI, which may impact embryo implantation.

## Introduction

Autoimmune thyroid disease (AITD) is one of the most common autoimmune disorders in women, which impacts 5%~15% women of childbearing age (1). Moreover, its prevalence is even higher in unexplained infertile women (14.5%–22.87%) (2, 3) and recurrent miscarriage (17.57%) (4). AITD is defined by increased thyroid autoantibodies with normal thyroid function and normal or slightly elevated thyroid-stimulating hormone (TSH) levels and lymphocyte infiltration in the thyroid. After immunosuppressive treatment, women with antithyroid antibodies undergoing assisted reproductive technology (ART) would manage to have an increased rate of clinical pregnancy (5, 6), which suggested that the correlation between AITD and unexplained infertility is potentially caused by the disruption of immune homeostasis. However, its pathogenic mechanism is beyond clarifying.

A successful implantation involves maternal immune tolerance to a semi-allogeneic embryo. During the window of implantation (WOI), dynamic changes of maternal immune cells may play a central role in the process of tissue renewal and differentiation and also participate in the transformation into a receptive endometrium (7). Previous studies have shown that uterine natural killer (uNK) cells are the major component of endometrial immune cells (8), which express lower cytotoxicity and exhibit the supporting ability of trophoblast invasion (9). T cells constitute the second largest group, and Th1-cell to Th2-cell transition improves the endometrial receptivity (10). Regulatory T cells (Tregs) are actively recruited along with neutrophils, dendritic cells (DCs), macrophages, and mast cells, into the endometrium during embryo implantation (11). Women with AITD tend to have dysfunctional T cells (12) and more NK cells (13) in peripheral blood. However, the maternal immune status and its potential effects on transcriptomic activation in the endometrium of AITD women during the WOI remain unclear. It is important to elucidate whether AITD represents an immune homeostasis disruption in the uterus which would disturb the crosstalk between embryo and maternal endometrium.

In this study, we used single-cell RNA sequencing (scRNA-seq) and collected endometrial samples from unexplained infertile AITD+ women with euthyroid function and AITD− women undergoing ART for male factors (oligospermia or azoospermia), to identify the immune cells and genes that are involved in endometrial receptivity to detect the underlying mechanism of AITD on women infertility firstly, and to evaluate the value of thyroid antibodies screening for unexplained infertility.

## Materials and methods

### Subject details and ethic permission

All donors between 18 and 34 years old have a comparable body mass index (18.5–23.9 kg/m^2^) and a normal karyotype. All women had regular menstrual cycles (3–4 days every 28–30 days) when the biopsies were taken, with no influence from exogenous hormones and medicine or gynecologic disease. They all exhibited a luteinizing hormone surge, and ultrasound scans were used to detect ovulation 36 h later. Then, endometrial biopsies were collected during 5–7 days after ovulation, and pathological examination was used to verify the phase of the menstrual cycle. In this study, the group of patients included three euthyroid AITD+ women with unexplained infertility, and euthyroid AITD− women were taken as the control (n=3, with male factors, two oligospermia and one azoospermia). To verify our results, we collected another three patients’ and five controls’ endometrium for flow cytometry (**Supplementary Table S1**). The study was reviewed and approved by the Ethics Committee of the Second Affiliated Hospital of Zhejiang University School of Medicine (2019-209).

### Cell isolation and sorting

Endometrial tissues were collected by a disposable biopsy device (Youjing, China). All samples were processed for single-cell digestion within 1 h after surgery. The endometrium was washed with ice-cold PBS and then trimmed into 1-mm^3^ pieces by ophthalmic scissors and digested in 3 ml of 1 mg/ml collagenase type IV (Sigma-Aldrich, C5138) and 0.01 mg/ml DNase I (Sigma-Aldrich, D5025) in DMEM (Gibco, C11995500BT)/10%FBS (Sijiqing, 13011-8611) with gentle shaking in a water bath for 45 min at 37°C. The supernatant was diluted with 3 ml of iced DMEM/10%FBS and passed through a 100-μm cell sieve (Corning, 431752) and then a 70-μm cell sieve (Corning, 431750). The flow-through was centrifuged and resuspended in 5 ml of red blood cell lysis buffer (Solarbio, R1010) for 10 min on ice. The cell suspensions were collected by centrifuging at 1,000 rpm for 5 min.

The endometrial leukocytes were purified by fluorescence-activated cell sorting (FACS) with 7-aminoactinomycin D (7-AAD; BD, 559925) and FITC-labeled anti-CD45 antibodies (BioLegend, 304005). Freshly purified endometrial leukocytes were immediately subjected to 10× scRNA-seq library preparation, as described below. For flow cytometric sorting, cells were stained with specific antibodies and isolated. Cell capture and analysis were performed on an MoFlo Astrios flow cytometer (Beckman Coulter). FlowJo 10 software was used for tSNE analysis.

The following antibodies were used for the analysis of endometrial immune cells with FACS, cell-surface staining, and cell sorting: FIXABLE VIABILITY DYE EF455 UV (Thermo); AF700 anti-human CD45, PE/CY7 anti-human CD16, PE anti-human NKp80, PE-CF594 anti-human CD11c, PE-Cy7 anti-human CD20, FITC anti-human CD4, BV510 anti-human CD8, and PE anti-human FGFBP2 (all from BioLegend); APC-Cy7 anti-human CD3, BV421 anti-human CD56, BUV615 anti-human CD117, BUV661 anti-human CD127, BB700 anti-human CD366/Tim-3, BV510 anti-human CD2, FITC anti-human CD103, BUV615 anti-human CD19, APC anti-human CD14, BUV661 anti-human HLA-DR, BV421 anti-human CD25, and BB700 anti-human CX3CR1 (BD Pharmingen).

### Single-cell RNA sequencing

Viable CD45^+^ cells from each subject purified by FACS were then resuspended at a concentration of 1,000 cells/μl, following the instructions of single-cell 3′ solution v3 reagent kit (10X Genomics) to capture around 10,000 cells per sample. The protoplast suspension was then loaded into Chromium microfluidic chips and barcoded with a 10X Chromium Controller (10X Genomics). RNA from the barcoded cells was subsequently reverse-transcribed, and sequencing libraries were constructed with reagents from a Chromium Single Cell 3′ v3 Reagent Kit (10X Genomics) according to the manufacturer’s instructions. Sequencing was performed with Illumina (NovaSeq 6000) according to the manufacturer’s instructions (Illumina). Each sample was sequenced for at least 150 Gb.

### Single-cell RNA-seq clustering analysis

We converted Illumina BCL files into fastq files using Cellranger mkfastq (https://support.10xgenomics.com/single-cell-gene-expression/software/pipelines/latest/what-is-cell-ranger). Then, we demultiplexed cells, aligned reads, and quantified gene-level expression using Cellranger count with default parameters. For the reference genome, we used the GRCh37 human genome. Potential doublets were detected using DoubletFinder (14) and removed. The number of statistically significant principal components (PCs) was set as 30, and the number of artificial doublets (pN) was set as 20%. The doublet rates were set as 0.061, 0.061, 0.054, 0.054, 0.069, and 0.054 for C1, C2, C3, P1, P2, and P3, respectively, according to their total cell numbers. After that, we processed integration and cluster steps as follows. First, genes expressed in at least three cells and cells with at least 200 genes expressed were kept.

Then, we calculated the interquartile range (IQR) of three features—number of RNA counts, percentage of mitochondrial gene expression, and percentage of dissociation-related gene expression (**Supplementary Table S2**)—and removed cells whose values of any of the features were higher by 1.5 times of IQR. After that, we calculated the mitochondrial mapping percentage, ribosomal mapping percentage, and cell cycle score with cell-cycle genes coming from Seurat’s cc.genes database (15). Then, we normalized and scaled our metadata by Seurat function SCTransform with a parameter vars.to.regress set to add scores we had calculated to the list of variables to regress, which can prevent those variables contributing much to the PCA. Cells were visualized with Uniform Manifold Approximation and Projection (UMAP) using 17 PCs.

To identify distinct cell types, especially subpopulations, we used custom codes modified from cellfindr, an algorithm that iteratively applies Louvain clustering to single-cell datasets in an automated fashion to identify biologically meaningful cell subpopulations (16). Cellfindr found variable genes using the Seurat FindVariableFeatures function with the default parameters. Then, PCs were constructed according to the variable genes. Cellfindr would choose the proper PC number automatically based on the combination of percent change in variation between the consecutive PCs and the contribution to the explanation for the standard deviation of each PC. The shared nearest neighbor graph was constructed using the determined PC number. The resolution parameter to find the resulting number of clusters was determined automatically by Cellfindr function find_res, which ensured that the maximum number of cell clusters being detected and enough significant marker genes exist for each cluster. Significant marker genes were detected by differential expression analysis based on the receiver operating characteristic (ROC) test. After the clusters were produced, cellfindr was used to perform the above process iteratively for each cluster to identify subclusters, or subclusters of subclusters, until no more subclusters could be found. UMAP analysis was performed using the RunUMAP function with default parameters. Clusters were annotated using canonical cell-type markers. Here, we identified all clusters with at least three differential expressed genes whose ROC value was larger than 0.6.

### Differential gene expression analysis

We performed the differential gene identification process by the Seurat “FindMarkers” command with default parameters. After that, we excluded all mitochondrial and ribosomal genes featured by initial MT- and RP characters in gene names. We performed further Gene Oncology (GO) enrichment with the package of genome-wide annotation for human org.Hs.eg.db through the R package clusterProfiler. Our differential gene analyses were divided into three parts: analysis for differential expression genes (DEGs) in the same maintype or subtype cell groups between control and patient samples, analysis for cluster-specific genes to illustrate group function, and analysis for high-frequency DEGs that recurred in more than a half of maintype groups (>5 maintypes) or subtype groups (>13 subtypes). For DEGs between control and patients, we split the differential expression gene lists we got into two parts by the sign of log2fold change (log_2_FC) values (log_2_FC > 1.0 and Bonferroni-adjusted P < 0.05) and did GO enrichment for them separately to distinguish function upregulated or downregulated in patients. For cluster-specific genes, we only kept to the analysis step for those genes with the positive log_2_FC sign, which means an additional function in a specific cluster. For high-frequency genes, we counted high-frequency upregulated genes, high-frequency downregulated genes, and high-frequency differential expression genes, then did enrichment for them all. In the GO enrichment steps, we set the *P* value cutoff to 0.01, the *q* value cutoff to 0.05. Then, we simplified synonymous GO terms with command “Simplify” with a cutoff p.adjust value set to 0.05.

### RNA velocity analysis

We did RNA velocity to identify the transition directionality of NK and T cells. After having produced loom files for every sample with command run10X in package version 0.17 velocyto.py according to reference genome GRCh38 with standard workflow (http://velocyto.org/velocyto.py/tutorial/cli.html#run10x-run-on-10x-chromium-samples), we exported UMAP cell embedding information, cells’ subtypes, and ID number for all uterine NK, CD8^+^ T cell, and CD4^+^ T cell subtype groups. Analysis of cellular trajectory by RNA velocity was performed with the Python package scVelo version 0.2.3 using stochastic modeling (17, 18) according to this pipeline (https://scvelo.readthedocs.io/VelocityBasics/). Genes with less than a sum of 30 unspliced and spliced read counts were filtered and the top 2,000 high-variation genes were kept in the preprocessing step with the “scv.pp.filter_and_normalize” command. In the step to compute moments for velocity estimation among nearest neighbors in PCA space, we selected the top 30 principal components and used the default 30 number of neighbors.

### Cell–cell communication analysis

We used the CellChat R package (19) to represent the cell–cell communication networks via ligand–receptor interactions. Based on the secreted signaling pathways and the precompiled human protein–protein interactions from the CellChatDB database (19), we followed the published workflow (https://htmlpreview.github.io/?https://github.com/sqjin/CellChat/blob/master/tutorial/CellChat-vignette.html, https://htmlpreview.github.io/?https://github.com/sqjin/CellChat/blob/master/tutorial/Comparison_analysis_of_multiple_datasets.html) with default parameters to identify potential cell–cell communication networks between different types of immune cells. We identified overexpression genes with a gene expression percentage cutoff (thres.pc) set to 0.1, a log_2_FC threshold set to 0.1, and a *P* value threshold set to 1. Then, we got patients’ upregulated(downregulated) L–R interaction subset featured with both ligand and receptor log_2_FC >0.25 (<−0.25). To illustrate our results better, we made some further adjustments as follows according to the original CellChat results. When comparing the overall information flow of each signaling pathway, we separately exported the control and patient groups’ basic plus statistic result of function RankNet and filtered out pathways whose P value >0.05 (which means not having a significant difference in this pathway between control and patients) and pathways whose neither patient or control scaled contribution was less than 1. For visually comparing cell–cell communication using the circle plot, we exported data of control and patients that had been managed in a way similar to that in visualization function netVisual_aggregate. After that, we kept contacts simultaneously satisfying: having top 75% strength in both control and patients and top 75% strongest the absolute value of control’s strength minus patients’.

### RNA isolation and quantitative RT-PCR

Total RNA was extracted using TRIzol reagent (TaKaRa, China) following the manufacturer’s instructions. Total RNA was converted to cDNA using the PrimeScript RT Reagent Kit with gDNA Eraser (TaKaRa, China) according to the manufacturer’s instructions. Trace amounts of RNA were extracted using RNeasy Plus Micro Kit (Qiagen, USA). RT-qPCR was performed using the Applied Biosystems 7500 Fast Real-Time PCR Systems and SYBR Green PCR Master Mix (TaKaRa, China) in a 20-μl reaction according to the manufacturer’s instruction. GAPDH was used as the reference for mRNA, and all primers for the RT-qPCR are shown in **Supplementary Table S3**. The qPCR protocol was as follows: 95°C for 10 min and 40 cycles of 95°C for 15 s followed by 60°C for 1 min. The relative expression levels were calculated using the equation N = 2-ΔΔCt.

## Results

### Immune cell types and compositional differences of the endometrium during the WOI between AITD patients and healthy controls

**Figure 1:**
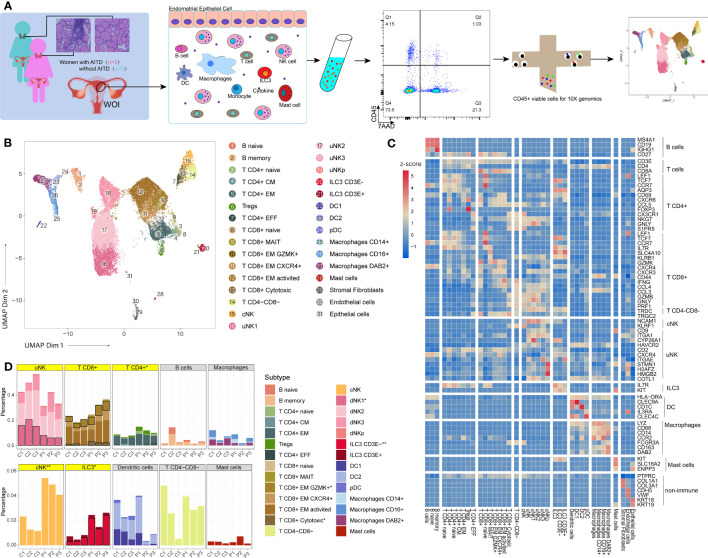
An atlas of endometrial immune cells in AITD patients and controls. **(A)** Flowchart depicting the overall design of the study. Numbers indicate the number of individuals analyzed. **(B)** A UMAP projection of the 26,875 total CD45^+^ leukocytes from three AITD patients and three controls, indicating 31 sub clusters. Different colors indicate different subtype cell clusters (see legend for key). **(C)** Heatmap of selected marker genes’ z-score for multiple immune cell subsets. The row shows the names of the endometrial cell clusters; the column represents the name of each gene. **(D)** Histogram showing the proportion (i.e., % of total sequenced immune cell complement) for each of the 11 main immune cell clusters and 28 subtype cell clusters in AITD patients and controls. The asterisk after maintype (in gray or yellow frame) or subtype names (in legends) means their proportion differences between health control and AITD patients are with statistical significance (*P* < 0.05; Student’s t-test); two asterisk means more distinctive statistical significance (*P* < 0.01; Student’s t-test). Significant subtype groups’ stacks are with black borders, whereas maintypes that have significant differences or contain significantly different subtypes are highlighted by yellow.

Our experimental procedure is shown in **Figure 1A**. Three AITD+ patients’ and three AITD− controls’ endometrial tissues were collected during the WOI. The detailed information of these subjects is shown in **Table 1**. Pathological examination of the endometrium presented mid-secretory manifestations (data were not shown). Using scRNA-seq, we identified 26,441 high-quality cells totally (13,332 cells from patients and 13,109 cells from controls) and 1,806 transcribed genes per cell (**Supplementary Table S4**). Our data showed good consistency in different groups (**Supplementary Figure S1**). The information of single-cell data before and after QC is presented in **Supplementary Figure S2**. There were 11 clusters identified, and then they were subdivided into 28 cell subsets (**Figure 1B**) according to the reported cell type or cell subtype markers (**Figure 1C**). Immune cells in the endometrium included NK cells, ILC3, T cells, DC, macrophages, B cells, and mast cells (**Figure 1B**; **Supplementary Figure S3**).

**Table 1:** Characteristics of subjects for scRNA-seq.

	Controls (*n*=3)	Patients (*n*=3)	*P*-value
Age	30 ± 2.65	28 ± 4	0.510
Body mass index (kg/m^2^)	20.39 ± 1.68	19.49 ± 0.69	0.440
TPOAb (IU/mL)	<0.5	536.50 (455.00)	0.037*
TgAb (IU/mL)	2.21 ± 1.31	330.32 ± 361.42	0.191
TSH (mIU/L)	1.53 ± 0.57	1.53 ± 1.10	0.997
FT4 (pmol/L)	13.11 ± 0.49	10.51 ± 5.79	0.480
FT3 (pmol/L)	5.12 ± 0.23	8.03 ± 6.16	0.460

*P < 0.05. Significance was evaluated with the Kruskal–Wallis test.

*FCGR3A*(CD16*)^-^ NCAM1*(CD56)^+^ cells were defined as uterine NK (uNK), which were the major part of endometrial immune cells and could be divided into four subtypes, namely, uNK1 (*HAVCR2* ^+^ *CYP26A1* ^+^), uNK2 (*CD2* ^+^), uNK3 (*CXCR4* ^+^ *ITGAE* ^+^), and uNKp (*STMN1* ^+^ *H2AFZ* ^+^ *HMGB2* ^+^ *COTL1* ^+^) (**Figure 1C**; **Supplementary Figures S4**; **S5**) (20). The conventional NK cell (cNK) group has a similar phenotype to the most general blood NK cells (*CD3E* ^-^ *FCGR3A* ^+^ *KLRF1* ^+^) (21). Moreover, ILC3 cells were defined as *KIT* ^+^ *IL7R* ^+^ (CD117^+^CD127^+^) (22), which were further divided into two subtypes based on their differential expression levels of *CD3E*: one subtype showed a higher transcription level (**Figure 1C**; **Supplementary Figure S6**) of *CD3E* gene (log_2_FC=1.47) than another group. There were 9,545 *CD3E^+^* T cells divided into 12 subsets (**Figures 1B, C**). These include five subsets of CD4^+^ T cells, six subsets of CD8^+^ T cells, and CD4^-^CD8^-^ T cells (γδ T) (*TRDC* ^+^, *TRGC2* ^+^). CD4^+^ T cells included naive CD4^+^ T cells (T CD4^+^ naive), central memory CD4^+^ T cells (T CD4^+^ CM), effector memory CD4^+^ T cells (T CD4^+^ EM), effector CD4^+^ T cells (T CD4^+^ EFF), and Treg cells. CD8^+^ T cells included naive CD8^+^ T cells (T CD8^+^ naive), effector memory CD8^+^ T cells (T CD8^+^ EM), T CD8^+^ EM CXCR4^+^ (which highly expressed *CXCR4*), T CD8^+^ EM GZMK^+^ (which highly expressed *GZMK*), activated central memory CD8^+^T cells (T CD8^+^ EM activated), cytotoxic CD8^+^ T cells (T CD8^+^ cytotoxic), and mucosal-associated invariant CD8^+^ T cells (T CD8^+^ MAIT) (23). DCs were defined as a combination of transcribed and non-transcribed genes (*HLA-DRA* ^+^ *NCAM1*(CD56)^-^ *CD14* ^-^ *CD19* ^-^ *CD3E* ^-^) (24), subtypes including *IL3RA*(CD123)^+^ *CLEC4C*(CD303)^+^ plasmacytoid DCs (pDC), *CLEC9A* ^+^ DC1, and *CD1C* ^+^ DC2. Macrophages were defined as *LYZ* ^+^ *CD68* ^+^ (25) and then were divided into three subtypes by the expression of *FCGR3A* (CD16) and *DAB2*, namely, macrophages CD14^+^, macrophages CD16^+^, and macrophages DAB2^+^ (26). Finally, we identified two B-cell subsets, namely, naive B cells (B naive) and memory B cells (B memory), based on the expression of *IGHG1*(IgG) (27). Mast cells were defined as *KIT*(CD117)^+^ *SLC18A2* ^+^ *ENPP3* ^+^ (**Supplementary Table S5**) (28).

Multiple cell types or subtypes showed a significant difference (*P* < 0.05, t-test, **Supplementary Table S6**) (**Figure 1D**). Compared with controls, AITD^+^ patients showed a significantly increased composition of CD4^+^ T, cNK, ILC3, *GZMK* ^+^ CD8^+^ T, cytotoxic CD8^+^ T, and *CD3E* ^-^ ILC3 cells whereas uNK1 cells significantly decreased. Meanwhile, uNK3 and CD8^+^ T cells increased slightly and uNK cells decreased slightly, which all showed no significant difference. We did not find compositional differences of DCs, B cells, and mast cells between the two groups. Therefore, we would focus on the differences of uNK, ILC3, and T cells between AITD patients and controls.

### *HAVCR2*(CD366)^+^ uNK1 cells were decreased in AITD patients, and DEGs revealed differences in NKs between two groups

**Figure 2:**
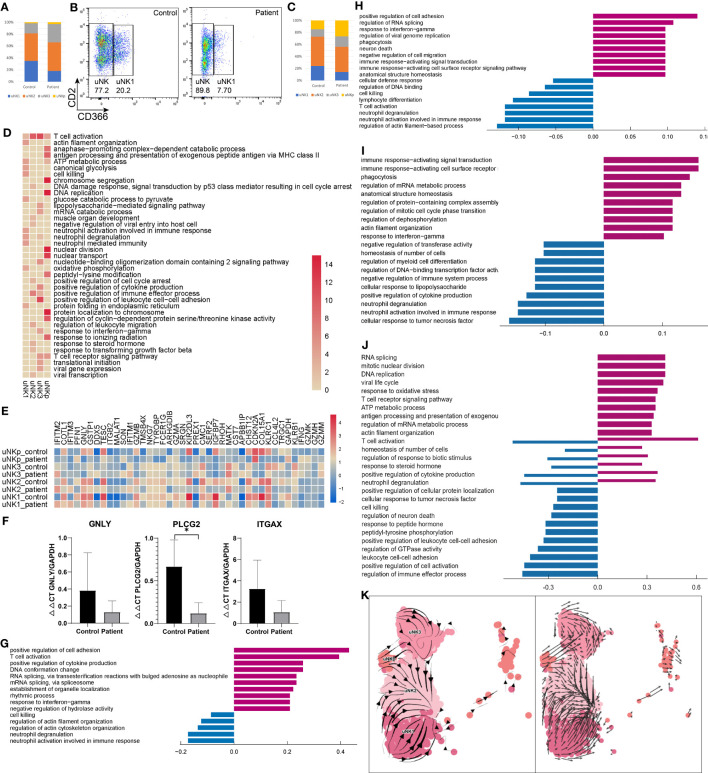
Characteristics of NK in the endometrium of AITD patients and controls. **(A)** The proportion of each uNK cell subset analyzed by scRNA-seq. **(B)** Flow cytometry showing the uNK1 cell subset. **(C)** The proportion of each uNK cell subset analyzed by flow cytometry. **(D)** Heatmap of the GO-enriched cluster-specific genomic features’ significance (-lg(p.adj); if >15, treated as 15) of different types of uNK cells. **(E)** Heatmap of DEGs’ z-score of each uNK cell type. **(F)** qPCR result showing the expression level of *GNLY*, *PLCG2*, and *ITGAX* in endometrial uNK2 cells. **(G-J)** Histogram showing differential top10 gene counts GO enrichment of each type of uNK in AITD patients and controls. uNK1 **(G)**, uNK2 **(H)**, uNK3 **(I)**, and uNKp **(J)**. Violet bars represent the upregulated pathways in patients, and blue bars represent the downregulated ones. **(K)** RNA velocity plotted in UMAP space for each uNK cell type. *P < 0.05. Significance was evaluated with Student’s t-test.

Four subpopulations of uNKs were identified in both AITD and healthy controls (**Figure 1B**). Relative decreases in uNK1 were noted in the AITD condition, whereas CXCR4^+^ uNK3 were slightly expanded (**Figure 2A**). Then, we performed flow cytometry to verify it; CD366^+^ uNK1 decreased in AITD (3.82 ± 1.41% vs. 7.79 ± 6.31%, *P*=0.388) (**Figures 2B, C**; **Supplementary Table S7**). The result without significant difference was caused by the limited sample size. Gene enrichment and pathway analysis showed that CD366^+^ uNK1 highly expressed *GSTP1*, *SPINK2*, *CD59*, *KIR2DL4*, *KIR3DL1*, and *FCER1G*, which reflected the function of uNK1 in cell process and cell killing (**Figure 2D**). uNK2 is characterized with high expression of *CD2*, which is the major type of uNK cells and accounts for ~40% of uNK cells (20% of endometrial immune cells) (**Figures 1B**, **2A, C**). *CD2* encodes protein that is reported to bind to LFA3 (CD58), inducing cellular adhesion, recruitment, and organizing of activating receptors to the immunological synapse (29, 30). Thus, uNK2 genes were enriched in regulating cytotoxicity, involved in antigen presentation (**Figure 2D**). Genes expressed in uNK3 were enriched in participating in chemotaxis and cell–cell adhesion (**Figure 2D**). Finally, uNKp, as the least population, was characterized by high expression of genes controlling translation machinery and cell-cycle events (**Figure 2D**), which indicated the differentiation potential of uNKp cells.

Differential expression analysis revealed upregulated and downregulated genes in each uNK subtype (**Figure 2E**; **Supplementary Table S8**). Notable upregulated genes were related to drivers of inflammation (*TNF*, *IFNG*), RNA processing, and transcription (*SON*, *DDX5*). Genes related to cell killing (*GNLY*, *GZMB*), chemotaxis (*CCL4L2*), degranulation (*ITGAX*), and lymphocyte differentiation (*PLCG2*) were downregulated (**Figure 2E**). qPCR also demonstrated that *GNLY*, *PLCG2*, and *ITGAX* were downregulated compared with controls in uNK cells (**Figure 2F**). GO analysis (**Figures 2G-J**) revealed increased expression of gene sets related to inflammatory response in uNK1, uNK2, and uNK3 cells and regulation of translation and cellular process in uNKp. In contrast, decreased expression of gene sets were related to degranulation and biological process in uNK1, uNK2, and uNK3 cells and cell–cell adhesion in uNKp. Additionally, uNKp response to inflammation and biological regulation showed a bidirectional regulation.

cNK cells account for a small part of endometrial immune cells. The proportion in patients was 4.82 ± 0.55%, which was significantly higher than that in controls (1.58 ± 0.53%, *P*=0.004) (**Figure 1C**; **Supplementary Table S6**). In flow cytometry results, the proportion of cNKs was 0.29 ± 0.12% vs. 0.44 ± 0.15%, which showed no significant difference (*P*=0.240) (**Supplementary Table S7**). The number of detected cNK cells in flow cytometry was much less than scRNA-seq results (0.39% vs. 3.2% on average), since the immune cell gating strategy in flow cytometry was different from scRNA analysis. cNK cells showed a higher expression of *KLRF1* in patients than controls, which can stimulate cNK cytotoxicity and cytokine release (31).

A previous study showed that decidual NKp cells differentiate and bifurcate into decidual NK3 cells (32). However, the differentiation of uNK without progesterone remains unclear. Here, we used RNA velocity (17, 18) to investigate the dynamic state among uNK1, uNK2, uNK3, and uNKp. In the RNA velocity results, the direction of state transitions and the extent of change in RNA dynamics are indicated by the vectors (arrows) and their lengths, respectively. We visualized the results in UMAP space by plotting an arrow for each cell, which spans the current and the inferred future states (**Figure 2K**). Within the uNK3 cell cluster, we observed short and uncoordinated RNA velocity, suggesting that these cells were in a transcriptionally stable state undergoing few changes. Arrows within the uNKp cells pointed toward either uNK3 or uNK2 cells, suggesting that these cells are possibly differentiated from the uNKp cells. Most arrows in the uNK1 cluster pointed away from uNK2, depicting the direction of the differentiation process. RNA velocity showed that uNK2 may separate from uNK1 and NKp, and uNK3 from uNKp, demonstrating distinct transcriptome signatures. These differentiation pathways between different uNK cells need further *in vitro* validation experiment in future.

### ILC3 increased in AITD patients and downregulated the extrinsic apoptosis signaling pathway

**Figure 3:**
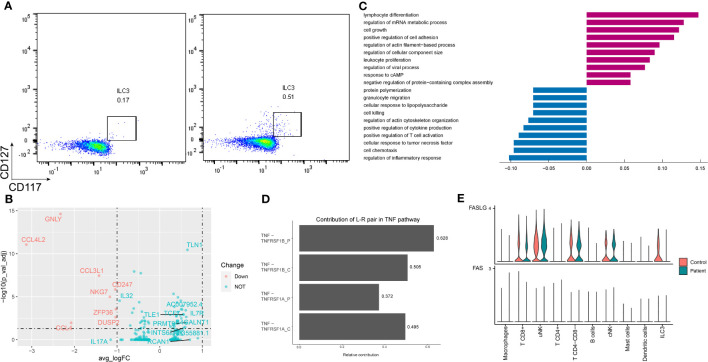
Characteristics of ILC3 in the endometrium of AITD patients and controls. **(A)** Flow cytometry showing ILC3 cells of control (left) and patient (right). **(B)** Volcano plot of DEGs in ILC3s. **(C)** Histogram showing immune-related differential genes’ GO enrichment of ILC3 in AITD patients and controls. **(D)** L–R pairs’ relative contribution separately in controls’ or patients’ TNF pathway from CellChat results. **(E)** Violin plots showing the normalized scRNA-seq expression distribution of FASLG-FAS pairs for control and AITD samples.

Two subpopulations of ILCs were identified according to the expression of *CD3E* (**Supplementary Figure S6**). While the ILC3 number was increased in the AITD condition, validated by flow cytometry (**Figure 3A**), ILC3 CD3E^-^ genes were enriched in leukocyte proliferation and ILC3 CD3E^+^ highly expressed genes were involved in cytokine production (**Supplementary Table S9**).

Differential expression analysis revealed 240 upregulated and 174 downregulated genes in the setting of ILC3 (**Figure 3B**; **Supplementary Table S8**). Genes controlling chemotaxis (*CCL3*, *CCL4*, *CCL5*), apoptosis (*FASLG*, *TNFRSF1A*), inflammation (*IL17A*), and cytotoxicity (*PRF1*, *IFNG*, *GNLY*) were among the top downregulated in the setting of ILC3. In contrast, genes regulating the regulation of translation (*EP300*, *STAT3*, *FOXP1*) were among the most heavily upregulated, although these genes showed no significant difference. GO analysis showed low expression of gene sets related to response to TNF, cell chemotaxis, and extrinsic apoptotic signaling pathway (**Figure 3C**).

The extrinsic apoptotic signaling pathway in ILC3 was induced by TNF-alpha and TNF receptor superfamily member 6 (FAS). Patients’ downregulated genes, including *ITM2C*, *GSTP1*, *TNFAIP3*, *TNFRSF1A*, *HSPA1A*, *FASLG*, *IFNG*, *HMGB2*, *CSF2*, and *IL2*, were all enriched in the extrinsic apoptotic signaling pathway (**Supplementary Table S10**). Most of them are related to apoptosis induced by TNF (33). To examine the impact of TNF family genes’ expression change in the patients on the cell–cell interactions, we further used CellChat to predict and compare the quantified ligand–receptor (L–R) interactions between the AITD patients and the healthy control. The contribution of *TNF-TNFRSF1A* to the cell–cell interactions was significantly reduced in patients (**Figure 3D**, *P*=0.034, Wilcoxon test with CellChat probability data), which triggered apoptosis or inflammation (34). In addition, in patients, among the FASLG pathway L–R pairs, ILC3s were predicted to lack the *FAS* signal (**Figure 3E**).

### T-cell subtypes showed difference between AITD patients and controls

**Figure 4:**
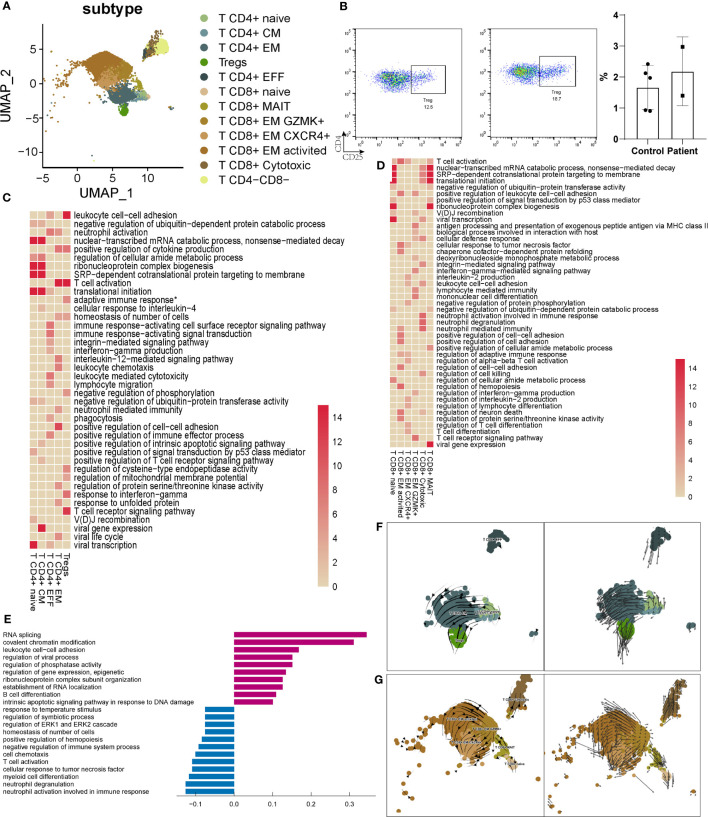
Characteristics of T cells in the endometrium of AITD patients and controls. **(A)** UMAP plots of 9545 T cells, indicating 12 clusters from AITD patients and healthy controls. **(B)** The proportion of Tregs analyzed by flow cytometry. **(C)** Heatmap of the enriched cluster-specific genomic features’ significance (-lg (p.adj); if >15, treated as 15) of different types of CD4^+^ T cells. Adaptive immune response* means adaptive immune response based on somatic recombination of immune receptors built from immunoglobulin superfamily domains. **(D)** Heatmap of the enriched cluster-specific genomic features’ significance (-lg (p.adj); if >15, treated as 15) of different types of CD8^+^ T cells. **(E)** Histogram showing differentially expressed genes’ top10 gene counts GO analysis of T CD8^+^ cytotoxic cells in AITD patients and controls. Violet bars represent the upregulated pathways in patients, and blue bars represent the downregulated ones. **(F, G)** RNA velocity analysis results of CD4^+^ T-cell subsets **(F)** and CD8^+^ T-cell subsets **(G)** in both healthy controls and AITD patients.

There were 12 subpopulations of T cells identified in AITD and controls (**Figure 4A**). While more CD4^+^ T and CD8^+^ T cells (T CD8^+^ GZMK^+^, T CD8^+^ cytotoxic) were present in AITD conditions (**Figure 1D**), the remaining proportions of subpopulations did not substantially differ. Treg is a specialized subpopulation of T cells that act to suppress immune response. *FOXP3* and *CD25* were defined as the gene markers of Tregs. The changes of number and function of Tregs could induce infertility and recurrent miscarriage (35). AITD patients showed more Tregs without significant difference (2.29 ± 1.08% vs. 0.87 ± 0.23%, *P*=0.145), which was supported by flow cytometry (2.04 ± 1.07% vs. 1.66 ± 0.63%, *P*=0.603) (**Figure 4B**; **Supplementary Table S7**). The result without significant difference was caused by the limited sample sizes. Genes expressed in Tregs were enriched in adaptive immune response and cell adhesion (**Figure 4C**; **Supplementary Table S9**). For GO analysis, upregulated genes in Tregs were enriched in cell–cell adhesion (*ROCK1*, *LGALS1*) and cell process (*SON*, *SRRM2*), whereas downregulated genes were enriched in cell–cell adhesion (*CCL3*, *CD40LG*) and lymphocyte migration (*CCL3*, *CCL4*). The imbalance of cell adhesion regulated by Tregs may induce impairment of pregnancy (**Supplementary Figure S7**).

For the other subtypes of CD4^+^ T cells (**Figure 4C**), T CD4^+^ naive and T CD4^+^ CM cells showed a similar phenotype, and genes expressed in these cells were enriched in cell differentiation. CD4^+^ T CM cells highly expressed genes were involved in cytoplasmic translation. Genes expressed in T CD4^+^ EFF were enriched in the “T cell receptor signaling pathway,” and CD4^+^ T EM in cell differentiation and immediate effector functions. Notably, upregulated genes in CD4^+^ T cells were related to cell adhesion (*IL7R*, *CTLA4*) and immune response (*TNFRSF4*, *TNFRSF1B*) (**Supplementary Figure S7**, **Tables S8**; **S10**), which may induce the increased number of CT4^+^ T cells in the endometrium. We found that *CCL4L2*, a gene showing the largest extent of expression difference between AITD patients and controls, was decreased for its expression in T CD4^+^ naive, T CD4^+^ EM, and T CD4^+^ EFF cells, although the function of this transcript remains elucidated. Other downregulated genes were involved in response to TNF (*TNFAIP3*, *NFKBIA*).

T CD8^+^ cytotoxic cells have the function associated with the signaling pathway of granulation (**Figure 4D**). *GZMB*, *GNLY*, and *PRF1* highly expressed in the T CD8^+^ cytotoxic cells were reported to specifically recognize HLA-I (36). The gene expression of T CD8^+^ cytotoxic cells showed that it had the same function of cNK cells in cytotoxicity. Upregulated genes related to immune response (*TNF*), RNA splicing (*SON*, *SRRM2*), and regulation of translation (*ATRX*, *ATM*). Downregulated genes were enriched in degranulation (*YPEL5*, *VAMP8*) (**Figure 4E**). Genes expressed in T CD8^+^ naive and T CD8^+^ MAIT cells were enriched in cellular process-associated GO terms (**Figure 4D**). T CD8^+^ EM GZMK^+^, T CD8^+^ EM CXCR4^+^, and T CD8^+^ EM activated cells expressed different cytokines. The genes expressed in them were also enriched in regulation of multiple biological functions. The DEG GO enrichment of the remaining types of T cells is shown in **Supplementary Figure S7**. T CD8^+^ EM-activated cells expressed more HLA-II (*HLA-DRB5*, *HLA-DQA2*) and increased the response to interferon-gamma (*IFITM2*). Downregulated genes in T CD8^+^ EM-activated cells were enriched in immune response (*CCL5*, *CD38*). In T CD8^+^ EM CXCR4^+^ cells, notable upregulated genes were related to cell adhesion (*ROCK1*, *TNF*, *LGALS1*); downregulated genes include those related to the immune response (*GZMB*, *KLRD1*, *KLRC2*). In T CD8^+^ EM GZMK^+^ cells, upregulated genes were enriched in the cell process (*FOSB*, *KLF6*), and downregulated genes in immune response (*FCER1G*, *IFNG*). T CD8^+^ naive and MAIT cells showed upregulation of translation and cell process (*SRSF1*, *SRSF11*). In contrast, decreased expression of gene sets was related to degranulation (*VAMP8*).

T CD4^-^CD8^-^ cells were a small part of T cells, which played an important role in immune response. Upregulated genes were enriched in the cell process (*SON*, *SRRM2*) and defense response to virus (*IFITM3*, *DDX17*), and downregulated genes in the metabolic process (*HSPA1A*, *HSPA1B*).

T cells in the different state of terminal differentiation showed various markers and performed different functions (37). CD4^+^ T cells could develop from naive T cells to T EM cells, T helper cells, or Treg cells separately, and CD8^+^ T cells develop from naive T cells to T EM cells (23, 38, 39). Thus, we performed RNA velocity analysis to analyze the differentiation dynamics in CD4^+^ T and CD8^+^ T cells. The CD4^+^ T CM and CD4^+^ T EFF states showed a low level of RNA velocities (short or no arrows), which is inferred to be associated with both quiescent and terminally differentiated cells, whereas the CD4^+^ T naive state exhibited a high level of RNA velocities. Increased arrow lengths marked the transitions from the CD4^+^ T naive state to the CD4^+^ T EM and Treg state and may reflect a rapid activation in RNA dynamics (**Figure 4F**). As for CD8^+^ T cells, the CD8^+^ T naive state with increasing arrow lengths reflects a rapid activation in RNA dynamics. The CD8^+^ T EM GZMK^+^ state may be quiescent and terminally differentiated cells with small RNA velocities. CD8^+^ T cytotoxic, CD8^+^ T EM activated, CD8^+^ T EM CXCR4^+^, and CD8^+^ T MAIT states exhibited large RNA velocities (**Figure 4G**).

### Characteristics of myeloid, B, and mast cells in AITD

**Figure 5:**
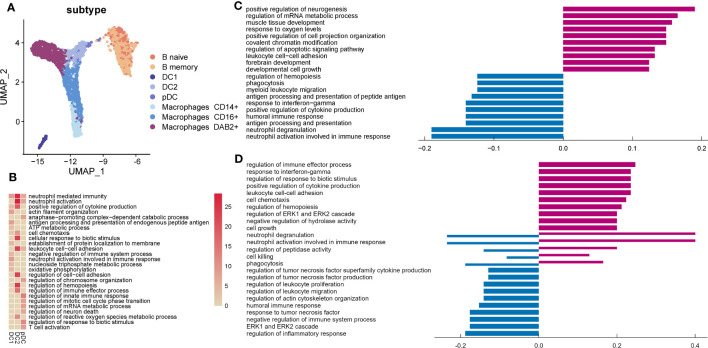
Characteristics of myeloid, B, and mast cells in the endometrium of AITD patients. **(A)** UMAP plots of myeloid, B, and mast cells from AITD patients and healthy controls. **(B)** Heatmap of the cluster-specific enriched genomic features’ significance (-lg (p.adj); if >15, treated as 15) of different types of DCs. **(C, D)** Histogram showing differentially expressed genes’ top10 gene counts GO analysis of DC2 **(C)** and macrophages **(D)**. Violet bars represent the upregulated pathways in patients, and blue bars represent the downregulated ones in AITD patients and controls.

From our UMAP plot, myeloid cells are clearly separated from others (**Figure 5A**). Genes expressed in DC1 were enriched in immune response, DC2 in antigen processing, and pDC in cell process (**Figure 5B**). DC2 expressed higher MHC II genes, such as *HLA-DQA2* and *HLA-DRB5* in AITD patients, and *GNLY* and *CCL4L2* were downregulated obviously (**Supplementary Table S8**). However, for GO analysis, upregulated genes in DC2 were related to the cell process (*FUS*, *HNRNPA2B1*) and downregulated genes were related to antigen processing (*HLA-C*, *HLA-DPA1*, *HLA-DMA*, *HLA-DQB1*, *HLA-DQA1*, *HLA-DRB1*) and response to interferon-gamma (*IFITM3*, *B2M*) (**Figure 5C**).

Genes expressed in macrophages CD14^+^ were annotated to inflammatory responses as part of the innate immune response to foreign pathogens. Genes expressed in macrophages CD16^+^ were related to inflammatory responses to viral infections, antigen processing, and metabolic process (**Supplementary Figure S8**). Macrophages DAB2^+^ played an important role in innate and adaptive immune response. In macrophages DAB2^+^, we found that *CCL3L1*, *CCL4L2*, and *GNLY* were significantly downregulated in the patients whereas *HLA-DQA2*, *MT2A*, *HLA-DRB5*, *MT1G*, and *FN1* were upregulated (**Supplementary Table S8**). From GO analysis, upregulated and downregulated genes were significantly associated with related genes of regulation of immune response (**Figure 5D**). Additionally, upregulated genes enriched in response to interferon-gamma (*IRF1*, *GBP2*) and cell–cell adhesion (*CD44*, *CD55*), and downregulated genes in response to TNF (*TNFSF12*, *TNFRSF1A*). Moreover, cell killing and cytotoxicity of macrophages showed variability.

B cells produce various kinds of immunoglobulin and participate in humoral immunity. B naive cells acted as a part of immune response, and B memory cells participated in recruiting immune cells for secondary immune response (**Supplementary Figure S9**). *HLA-DQA2* was increased in B cells of patients, whereas *GNLY* and *CCL4L2* were significantly decreased (**Supplementary Table S8**). It was reported that *HLA-DQA2* was expressed in B lymphocyte functions in delivering antigenic peptides to CD4^+^ T cells (40). According to GO analysis, upregulated genes of B cells were enriched in the cell process (*SON*, *DDX5*) and activation (*LYN*, *PTPN6*), whereas downregulated genes were enriched in regulation of cytokine production (*TYROBP*) (**Supplementary Figure S10**).

The function of mast cells, a small part of immune cells in the endometrium, during embryo implantation remains to be determined (41). *CCL2*, *XCL2*, *XCL1*, and *TNFAIP3* were downregulated in mast cells (**Supplementary Table S8**), which were annotated to “response to tumor necrosis factor” (**Supplementary Figure S11**), whereas there were no significantly upregulated genes.

### Changes in cell–cell interactions between the AITD patients and the healthy controls

In the endometrium during the WOI, immune cells showed different states and probably different cell–cell interactions between patients and controls. The CellChat database (19) was used to analyze cell–cell communication for detailed changes in terms of cell types and signaling pathways or ligand–receptor pairs between patients and controls.

**Figure 6:**
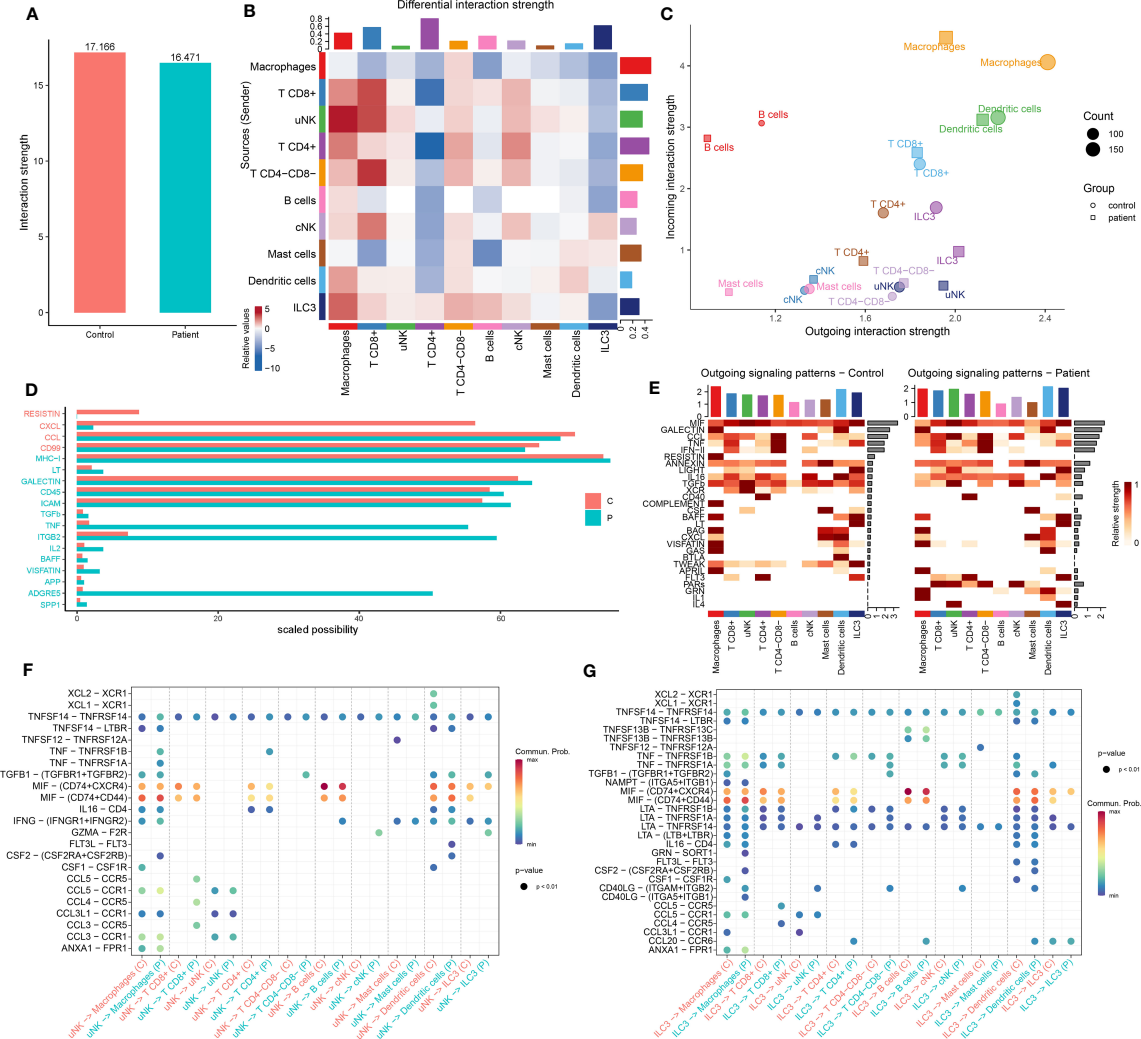
Comparison of CellChat in immune cells between AITD patients and controls. **(A)** Total interaction strength in control and AITD groups. **(B)** Heatmap of differential interaction strength; different colors in the border represent different cell groups. X-axis means signal receiver, and Y axis means signal sender. Red means increased signaling pathways, and blue means decreased signaling pathways. Histograms in the column mean totally sent number of interactions/interaction strength of every group, whereas histograms in the row mean totally received number of interactions/interaction strength of every group. **(C)** Magnified view of each cell group’s total incoming or outgoing strength. Circle and square symbols represent the signaling networks of control and patient, respectively. Different colors represent different groups of immune cells. X-axis means total received signal strength, Y-axis means total sent signal strength. **(D)** Information flows of scaled probabilities within the inferred networks between AITD patients and controls; part of them (scaled probability > 1 in patients or controls; *P* < 0.05, paired Wilcoxon test) were shown here. The red color means health controls’ information flow whereas green means patients’. The full name of these pathways is shown in **Supplementary Table S11**. **(E)** Contribution of the pathways identified to the outgoing signals among the different cell types in control (left) and AITD patients (right). **(F, G)** Bubble plot of the communication probability of all the significant ligand–receptor pairs that are sent from uNK **(F)** and ILC3 **(G)** cells in control and AITD groups.

Our analysis revealed less intercellular communication probability, represented by interaction strength, in the AITD group compared with the controls (**Figure 6A**). Macrophage was the dominant signaling source in the AITD condition, and T CD4^+^ cell and ILC3 were the dominant signaling targets (**Figure 6B**). Signals from uNK to macrophage, T CD8^+^, uNK, and cNK cells also increased in AITD conditions. In contrast, signals sent from uNK to T CD4^+^ cells and ILC3 were decreased. In a two-dimensional plot (**Supplementary Figure S12**), T CD4^+^, ILC3, and macrophage cells showed the largest difference between the two groups according to the shared two-dimensional manifold of their functional similarity. The differences of T CD4^+^, ILC3, and macrophages between AITD patients and controls resulted from incoming and outgoing signals jointly, whereas outgoing signals contributed to the differential interactions of uNK cells in two groups (**Figure 6C**).

Our result identified 107 total pathways among the 11 cell groups (**Supplementary Figure S13**). We found a significant decrease in the number of interactions in RESISTIN and CXCL pathways in patients (**Figure 6D**). The RESISTIN signaling pathway did not exist in patients, which is due to the decreasing expression of *RETN*. Meanwhile, our study suggested a potential increase in TNF, ITGB2, and ADGRE5 signaling in patients (**Figure 6D**), which has been shown to play a key role in inflammatory response and mediate the migration of leukocytes (42).

As the largest number of immune cells in the endometrium during WOI, we focused on the signals sent from uNK cells. uNK sent more CCL, TNF, IFN-II, IL16, LIGHT, FLT3, PARs, and IL4 signals and less CSF, XCR, and TWEAK signals to other cells (**Figure 6E**). Analysis of outgoing CCL and TNF signaling from uNK cells to macrophages and T CD8^+^ revealed several ligand–receptor pairs with high communication probabilities, including CCL3-CCR1, CCL3-CCR5, CCL4-CCR5, CCL5-CCR1, CCL5-CCR5, TNF-TNFRSF1B, and TNF-TNFRSF1A (**Figure 6F**). ILC3 expressed lower *CCL3*, *CCL4*, *CCL5*, and *CCL20*. However, signaling from ILC3 to other cell types showed high communication probabilities in several ligand–receptor pairs (**Figure 6G**).

## Discussion

Embryo implantation occurs during the WOI; an elaborate immunomodulation was observed in this period (43). In this study, we clarified the difference of immune cells in the endometrium during the WOI stage between AITD^+^ patients and AITD^-^ controls for the first time via scRNA-seq analysis. AITD patients exhibited decreased CD366^+^ uNK1 cell number and an increased number of CD4^+^ T, cNK, ILC3, T CD8^+^ GZMK^+^, T CD8^+^ cytotoxic, and ILC3 CD3E^-^ cells. CXCR4^+^ uNK3 cells were slightly increased. Each type of immune cells showed several DEGs, particularly the expression level of *GNLY* was decreased significantly in NK cells. *CCL3L1* and *CCL4L2* were decreased in several cell types. DEGs in uNK and CD4^+^ T cells showed the activation of immune response. uNK cells showed decreased cytotoxicity, whereas cNK worked with increased cytotoxicity. There was a rare population of ILC3 cells in the endometrium, but they played a crucial role in innate immunity and were predicted to have inhibited the extrinsic apoptosis signaling pathway in the patients through TNF, which may explain the increased number of ILC3 in the AITD endometrium. The slightly increased Tregs showed a bidirectional regulation of cell–cell adhesion. Upregulated genes in T CD8^+^ cytotoxic cells were enriched in RNA splicing, but degranulation-associated genes were downregulated. uNK cells sent the dominant outgoing signals, and increased CCL and TNF signaling pathways played an important role during WOI.

Previous studies favor a significantly increased incidence of infertility among euthyroid AITD women. Infertile patients with AITD who received ART suffered from lower pregnancy rates per initiated cycle and per embryo transfer cycle (44); however, the oocytes retrieved and embryos transferred showed no significant difference compared with AITD^-^ controls. Randomized controlled studies (RCTs) demonstrated that the use of levothyroxine had no improvement on ART outcomes in infertile AITD patients (45, 46), which suggested that AITD-associated infertility is a non-endocrine autoimmune disease. In addition, immunosuppressants can improve pregnancy outcomes (47), but the treatment remains controversial, which may induce pregnancy complications (48, 49); it even manifests reproductive toxicity in an animal experiment (50). It has been shown that the number of certain types of immune cells was changed in peripheral blood of AITD patients (12, 13, 51), and Treg cells were increased in their endometrium (52). Owing to the limitation of methods, previous studies showed no correlation between AITD and the cytotoxicity of macrophages, DCs, T cells, and NK cells in the endometrium (52). Recently, scRNA-seq provided a new strategy to detect the cell–cell interaction between maternal immune cells and the transcriptomes of individual cells in the endometrium during WOI. The immunity imbalance in the microenvironment may interfere with endometrial receptivity and embryo implantation.

During early pregnancy, CD56^bright^ uNK cells infiltrate and accumulate around spiral arteries and participate in the regulation of placentation and trophoblast invasion (53). Pregnancy loss may be caused by either insufficient or excessive NK cell recruitment to the endometrium, secondary to dysregulated cytokine signaling (54). Our results showed that a decrease of CD366^+^ uNK1 cells, which displayed immunosuppressive activities, produced more anti-inflammatory cytokines than CD366^-^ NK cells and promoted inducible Treg differentiation (55). Moreover, dysregulation of CD366 expression can induce excessive or inhibited inflammatory responses and lead to autoimmune diseases (56). CD366^+^ NK cells are beneficial to pregnancy immunotolerance. The imbalance of the NK-cell proportion may destroy the local immune microenvironment.

The most remarkable DEG in uNK is *GNLY*, which was significantly downregulated in AITD patients. *GNLY* is expressed mainly in activated NK cells and plays an important role in activating the immune system in response to pathogens (57). It also induced apoptosis of extravillous trophoblasts (58) and activated T lymphocytes to stimulate mitogenicity of endothelial cells inducing angiogenesis (59). Human decidual NK cells highly express GNLY and selectively kill pathogens without killing the trophoblast in early pregnancy (60). The dysregulation of *GNLY* in uNK cells may interfere with the signal inhibitory interactions between NK cells and trophoblasts, inducing perforin recruitment and potentially harmful cytotoxicity (61). GNLY is not expressed in mice; GNLY-humanized mice are useful to detect its roles in regulating embryo implantation. Increased probabilities of signaling from uNK to other cells were associated with TNF. TNF signaling pathways participated in trophoblast biology, especially migratory activity, syncytialization, and endocrine function (62). TNFRSF1A and TNFRSF1B polymorphisms were a double-edged sword, TNFRSF1A predominantly triggered apoptosis or inflammation, and TNFRSF1B promoted tissue repair and regeneration (34). The expression of TNFRSF1A and TNFRSF1B attenuated the interaction between uNK, macrophage, and T CD8^+^ cells.

ILC3 is a small part of endometrial immune cells, which participate in innate defense mechanisms on mucous membranes (63). Uterine ILC3s play a preponderant role in embryo implantation and have an antimicrobial effect (64–66). Defects in ILC generation or functional interactions in decidual tissues may cause fetal loss (67). The ILC3 number has been shown to be increased in the thyroid tissues of patients with HT (68), similar to that in the endometrium we found. From the GO analysis, the extrinsic apoptotic signaling pathway induced by TNF was predicted to be inhibited in AITD patients, which may induce the increase of localized ILC3. Previously, ILC3 was divided into NCR^+^ and NCR^-^ subsets (66). NCR^-^ ILC3 could change phenotypic and functional features (67); in our study, NCR^-^ ILC3 has a thumping majority and the difference in *CD3E* expression was shown in two subtypes of ILC3. It needs further research that whether this method is universal in other tissue. ILC3 had a lower expression of *CCL4L2*, *CCL4*, and *CCL3L1* in AITD patients. *CCL4L2* and *CCL3L*1 have an additional copy number variations of CCL3 and CCL4 genes and show the same properties of CCL3 and CCL4 (69). ILC3 induces immune cells tumor infiltration (70). CCL3 and CCL4 can recruit downstream immune cells (71, 72) and regulate proinflammatory process (69). The probabilities of CCL signaling from ILC3 to other cell types increased in AITD. The probability score based on differentially co-expressed receptor–ligand pairs, which hinted the chemotaxis of ILC3 for B cells, DCs, T CD4^+^, and T CD8^+^ cells.

T cells are the second dominating immune cells in the endometrium, which have been increased in number in women with AITD. Tregs establish and maintain maternal–fetal immune tolerance, which increased in the endometrium of women with recurrent miscarriage (52), and Tregs also increased in AITD patients from our data. Co-localization of Tregs and ILC3s in the cryptopatches of the intestine has been observed (73). ILC3s may be selected for antigen-specific RORγt^+^ Treg cells, and against T helper 17 cells, to establish immune tolerance (74). The causal link between Tregs and ILC3 in the endometrium requires to be elucidated. The number of CD8^+^ T cells is almost two- to threefold compared with CD4^+^ T cells in the endometrium during the WOI. CD8^+^ T cells as a pivotal mechanism of immune homeostasis and tolerance are tilted in pathological contexts (75), such as preeclampsia (76), miscarriage (77), and intrauterine growth restriction (76). We found the increasing CD8^+^ T cytotoxic cells, which highly expressed *GNLY*, *GZMB*, and *PRF1*. T cells expressed enhanced levels of these genes that function in a cooperative manner to perforate the target cell membrane and initiate apoptosis (78) and participate in the proinflammatory process (79). Some previous papers revealed that decidual CD8^+^ T cells of healthy pregnant women do not express *GZMB* and *PRF1 (* 80, 81). However, *GZMB* was overrepresented in peripheral blood T cells from HT patients and its levels varied by age, thyroid volume, and disease severity (82). Elevated *GZMB* in preeclampsia inhibits invasion and migration of trophoblasts (83). PRF1 is a main mediator of cytotoxicity in decidua, which is required for the delivery of granzyme to the cytoplasm of the target cell (84). Thus, CD8^+^ T cytotoxic cells may get involved in the immunity homeostasis and vascular remodeling in the maternal–fetal interface.

The interaction between immune cells constitutes a vast network of cellular connections, which play an important role in embryo implantation during WOI. Thus, we use CellChat to analyze this interaction between immune cells. The RESISTIN and CXCL signaling pathways were significantly inhibited in AITD patients. The serum RESISTIN interaction level was decreased in untreated AITD patients and induced the decreasing neutrophil counts (85), which may impact the process of embryo implantation. CXCL signaling pathways induce lymphocyte migration (86), maintain immune tolerance (87), regulate trophoblast migration, invasion, and proliferation (88), positively affect endothelial cell proliferation (89), and stimulate decidual angiogenesis (90). The downregulation of the CXCL signaling pathway plays a negative role in modulating endometrial immune cells, trophoblasts, and epithelial cells. ITGB2 and ADGRE5 pathways were significantly increased in information flow. Integrin, as a cell surface receptor, mediates the adhesion of cells and the extracellular matrix. Its special type can also induce cell–cell interaction during the process of leukocyte adhesion. The mRNA level of *ITGB2* was decreased significantly in women with recurrent implantation failure (91), which revealed the effect of *ITGB2* in embryo implantation. ADGRE5 promoted trophoblast invasion via the PI3K/Akt/mTOR signaling pathway, which was decreased in preeclampsia patients (92). We came to the conclusions diametrically different from others; however, our result did reflect interaction strength but not the expression levels of genes. Immunoregulation was a complex process; whether a bidirectional regulation present in the maternal–fetal interface is worthy of further research.

In conclusion, our study provided the first immune atlas of unexplained infertile women with AITD during WOI, showing that AITD can disrupt immunity homeostasis in the endometrium, which may interfere with local microenvironment immune balance and induce changes of gene expression. The associated signaling pathway including cell apoptosis, chemotaxis, and cytotoxicity may influence endometrial receptivity. Physicians should attach more importance to recognize that immune disruption does exist in AITD^+^ women before embryo implantation. Further research remains in the methods of reversing the negative impact of AITD through interfering with potential pathogenic genes. Our research has certain limitations; based on ethics, when the endometrium was collected before embryo invasion, it was difficult to estimate the effect of embryo.

## Data availability statement

The datasets presented in this study can be found in online repositories. The names of the repository/repositories and accession number(s) can be found below: https://ngdc.cncb.ac.cn/, HRA003204.

## Ethics statement

The studies involving human participants were reviewed and approved by Ethics Committee of the Second Affiliated Hospital of Zhejiang University School of Medicine. The patients/participants provided their written informed consent to participate in this study.

## Author contributions

MJ, QZhou, JX, and AG designed the research, analyzed and interpreted data, and wrote the manuscript. HH, QZhao, YY, JC, ZC, and PZ performed research and collected, analyzed, and interpreted data. JX collected data, performed flow cytometry, data analysis, and manuscript preparation. AG performed bioinformatics, data analysis, and manuscript preparation. HH performed sample annotation, data analysis, data interpretation, and manuscript preparation. All authors contributed to the article and approved the submitted version.
