# Supplementary material for: Autoimmune thyroid disease disrupts immune homeostasis in the endometrium of unexplained infertility women—a single-cell RNA transcriptome study during the implantation window
Source: Front Endocrinol (Lausanne). 2023 Jul 12;14:1185147. doi: 10.3389/fendo.2023.1185147 (PMC10368980; doi:10.3389/fendo.2023.1185147)
Supplement: Supplementary file 1 [file Image_1.pdf]

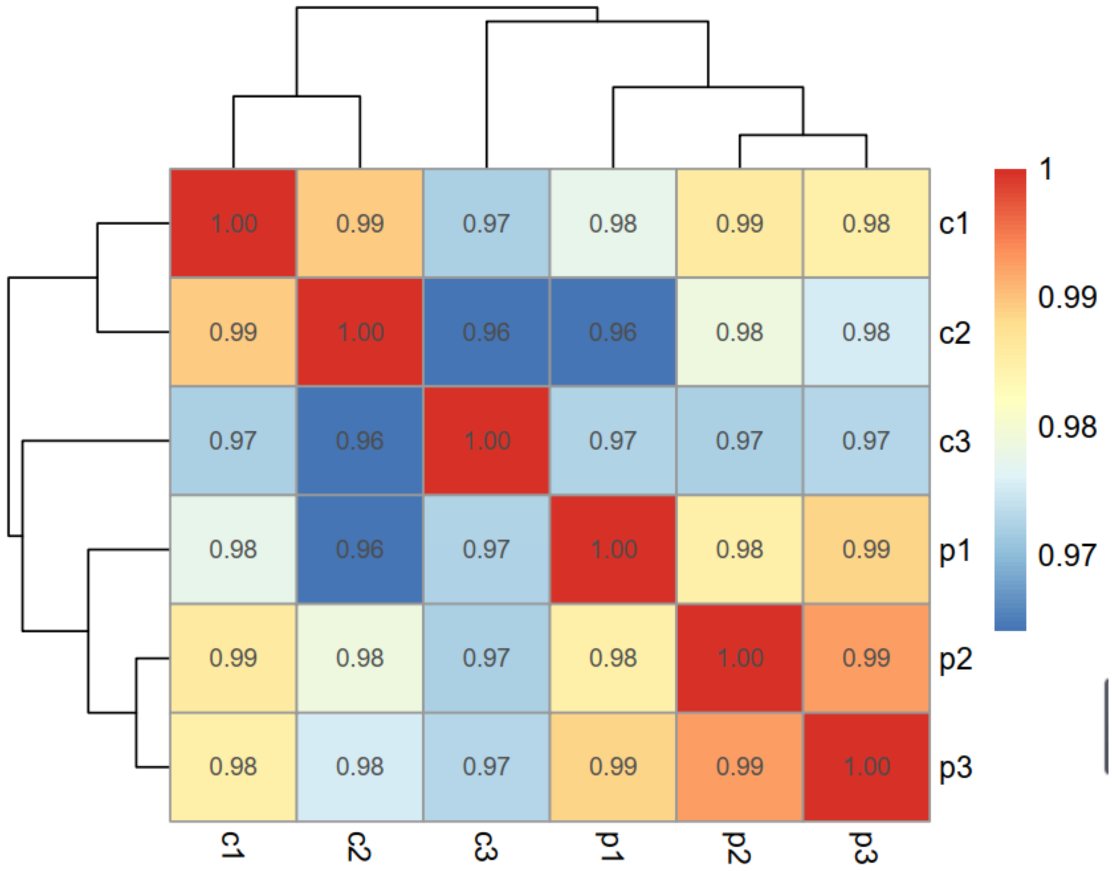

Figure. S1 Heatmap of correlation in different groups.

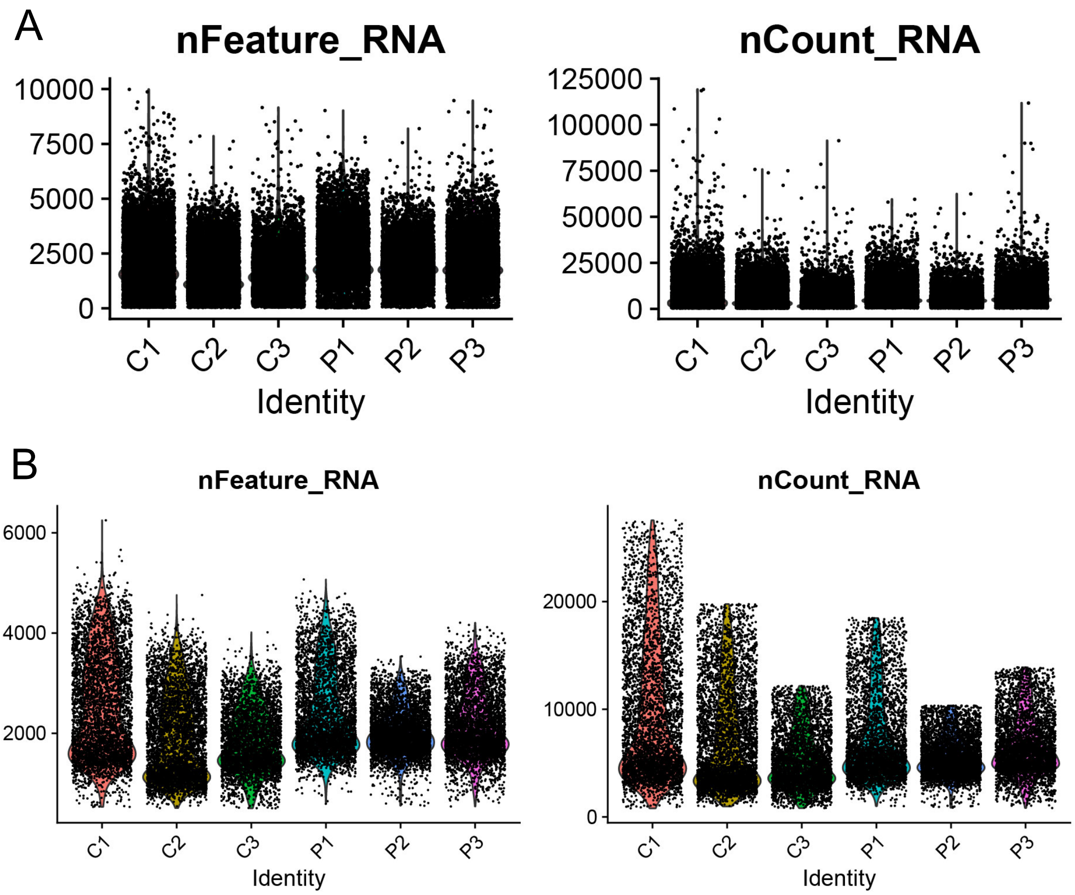

Figure. S2 scRNA-seq quality control plots. (A) Number of genes (nFeature_RNA) and Number of UMIs (nCount_RNA) per sample before filtered. (B) Number of genes (nFeature_RNA) and Number of UMIs (nCount_RNA) per sample after filtered.

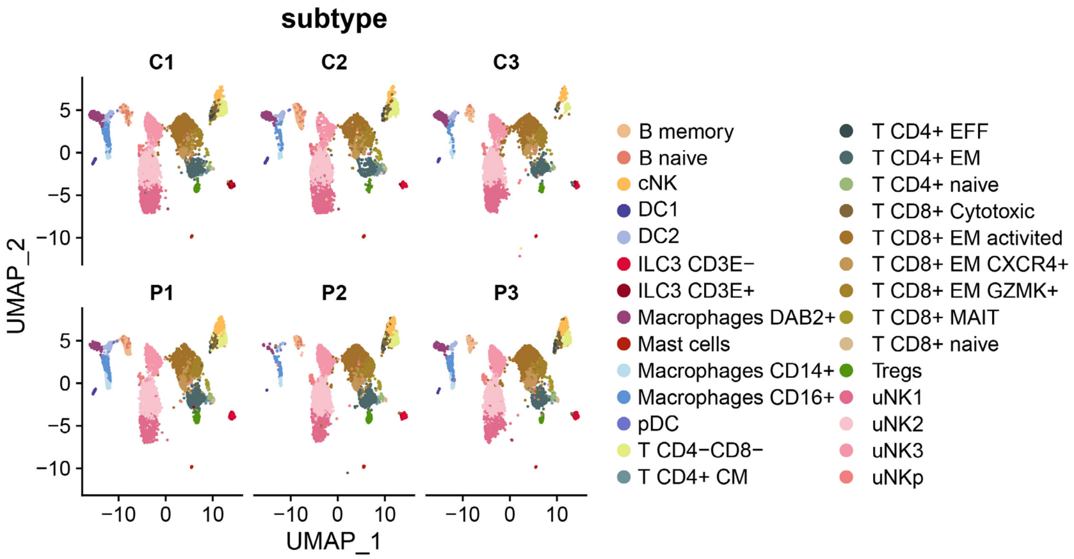

Figure. S3 UMAP plots of total CD45+ leukocytes from 3 AITD patients and 3 controls, indicating 31 clusters. Different colors indicate cell clusters (see legend for key).

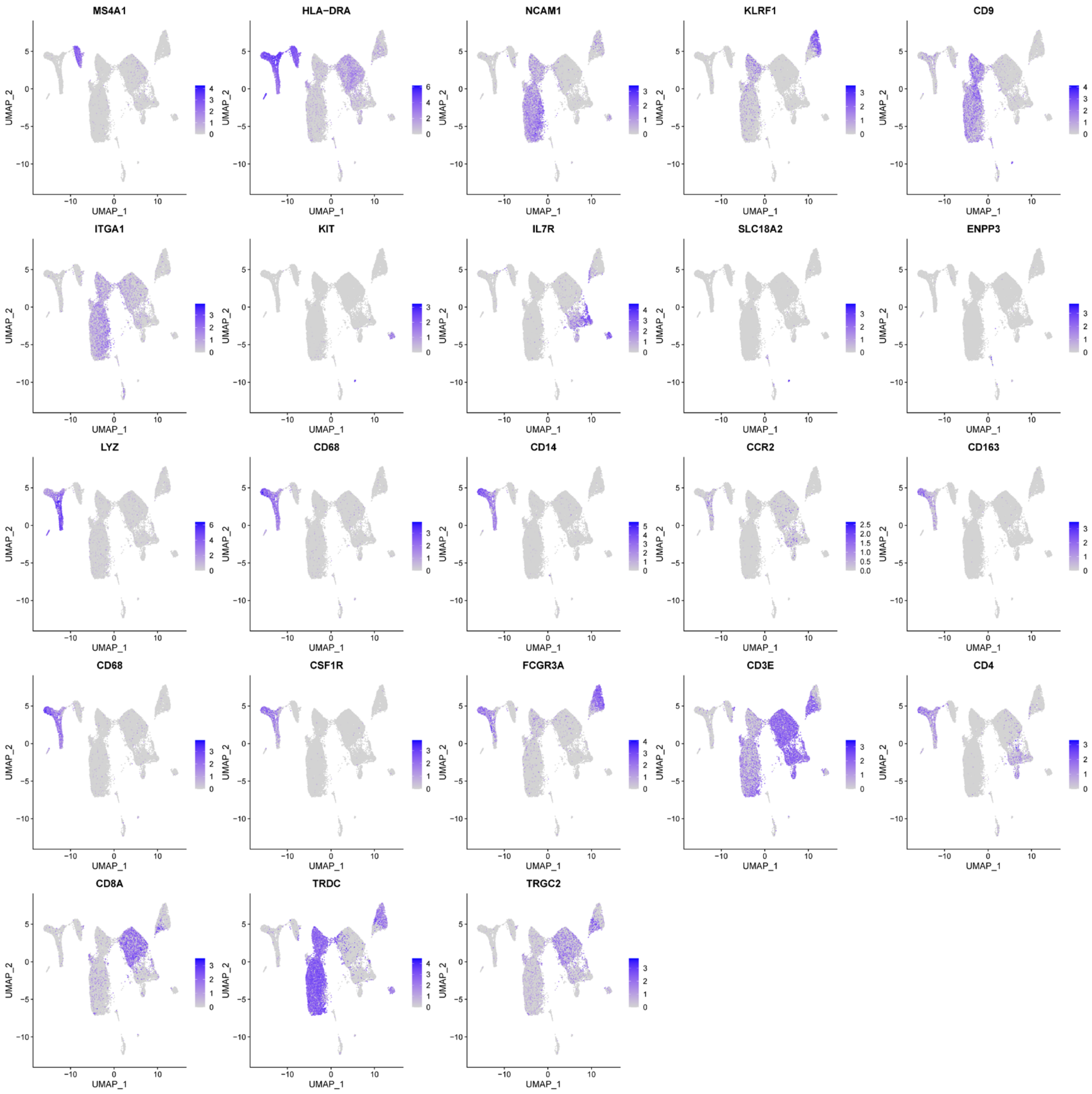

Figure. S4 UMAP plots of cell markers expression for annotation.

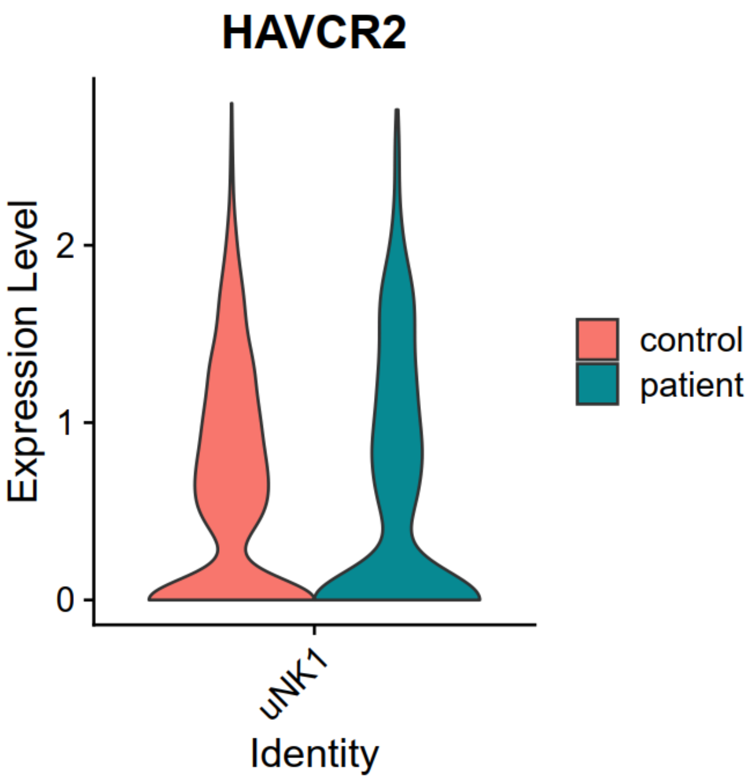

Figure. S5 Violin plot of CD366 (HAVCR2) expression in uNK1 cells.

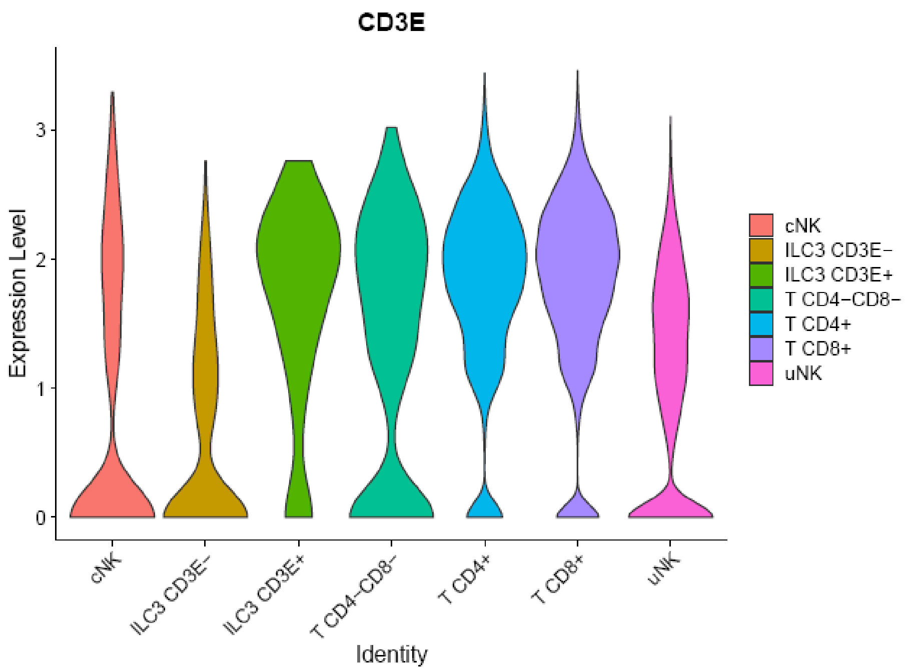

Figure. S6 Violin plot of CD3E expression in several cell types.

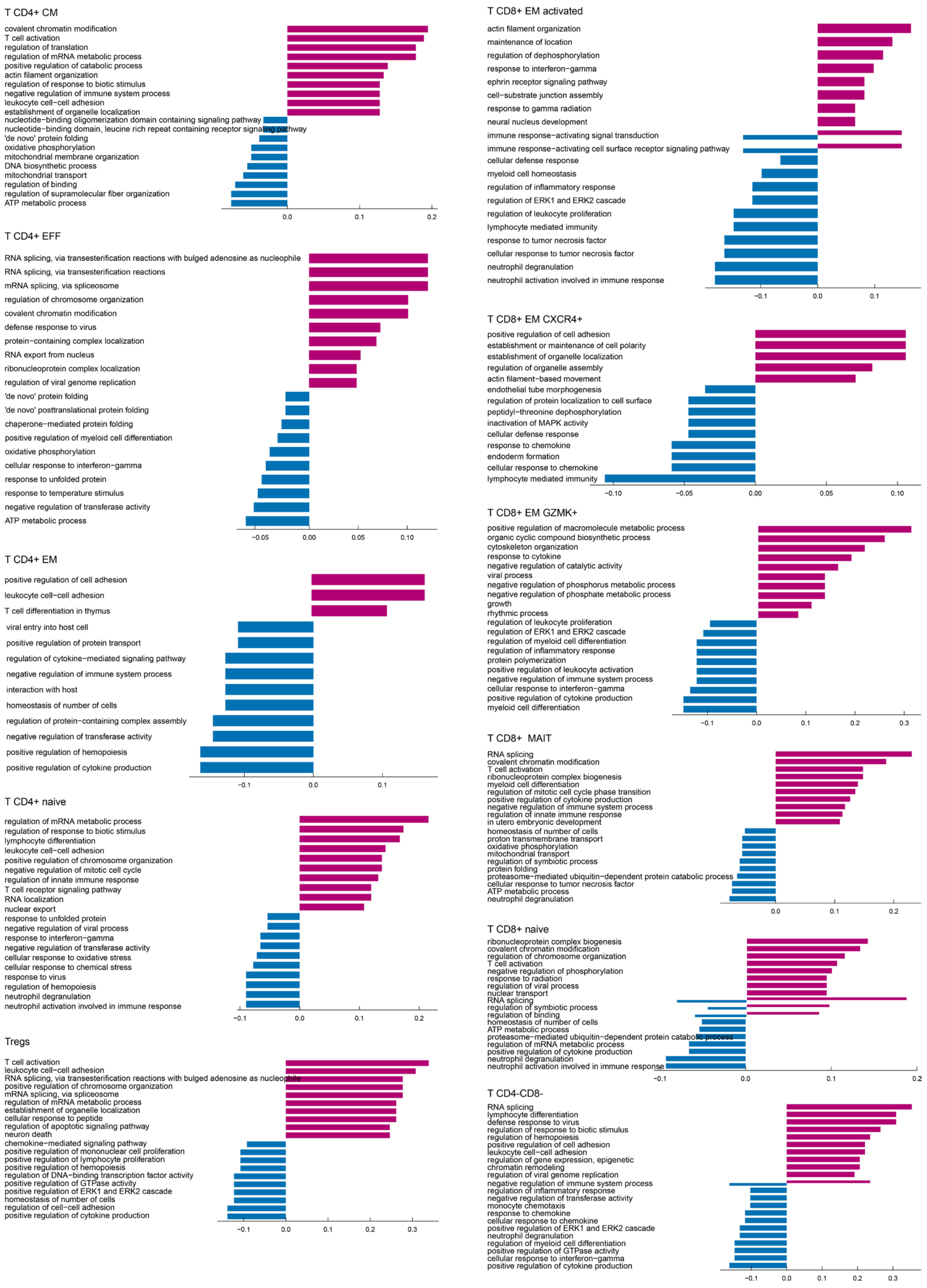

Figure. S7 Differences expressed genes' GO result. Showing the patient up-regulated or down-regulated hallmark pathways of each T cell subset. Violet bars represented the up-regulated pathways in patients, and blue bars represented the down-regulated ones.

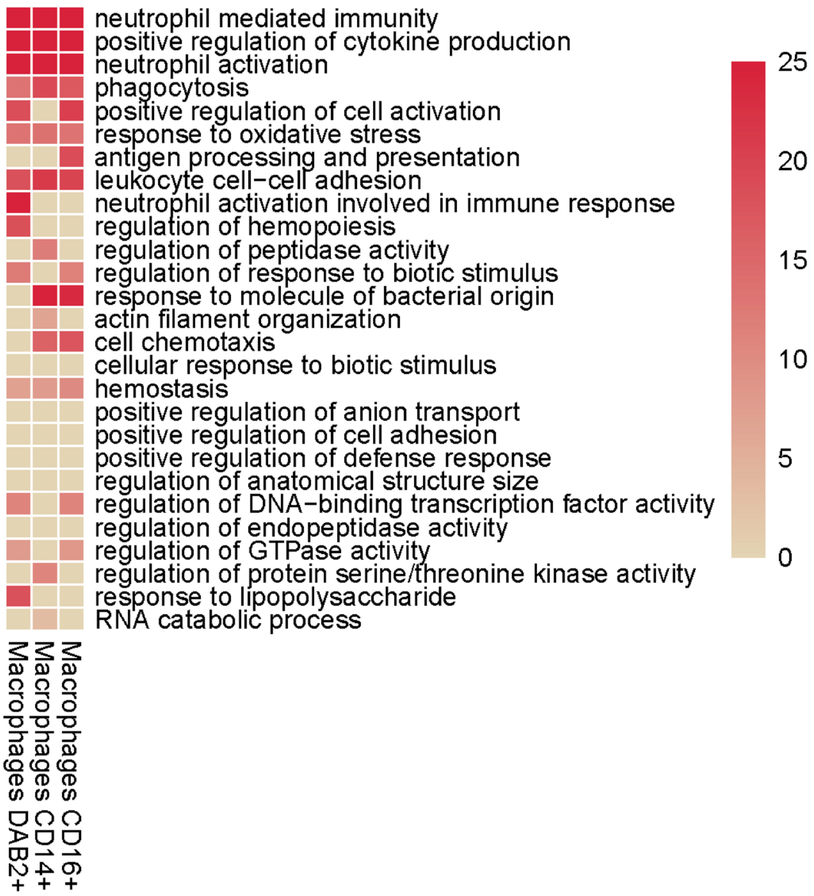

Figure. S8 Heatmap of the enriched cluster specific genomic features' significances (-lg(p.adj); if > 15, treated as 15) of different types of Macrophages.

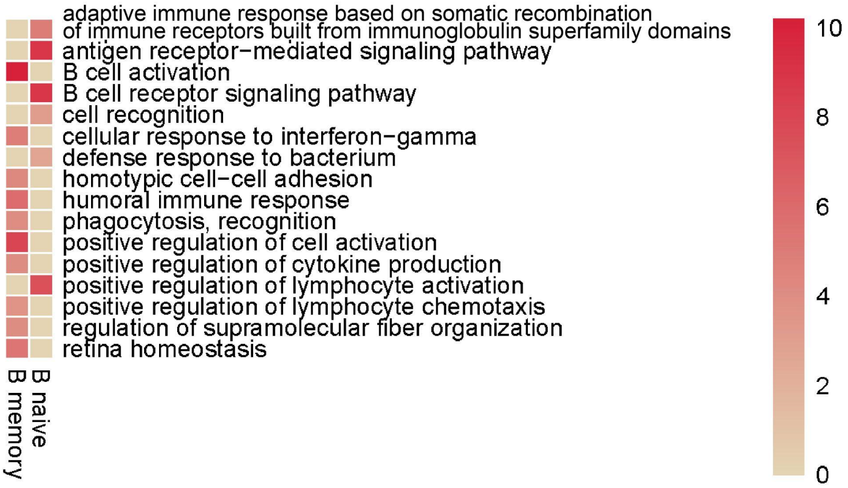

Figure. S9 Heatmap of the enriched cluster specific genomic features' significances (-lg(p.adj); if > 15, treated as 15) of different types of B cells.

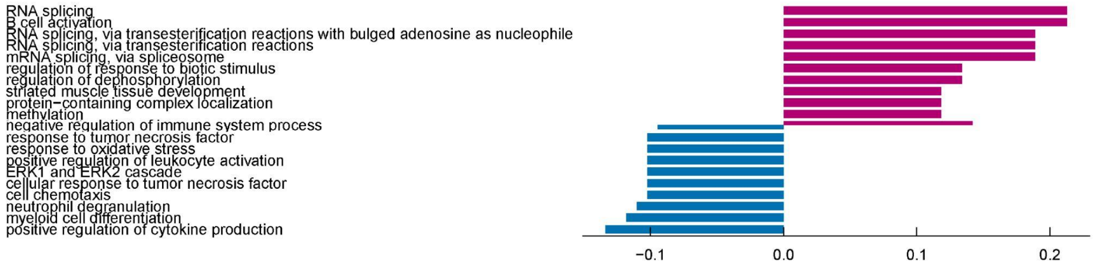

Figure. S10 Histogram showing differentially expressed genes' GO enrichment of B cells in AITD patients and controls. Violet bars represented the up-regulated pathways in patients, and blue bars represented the down-regulated ones.

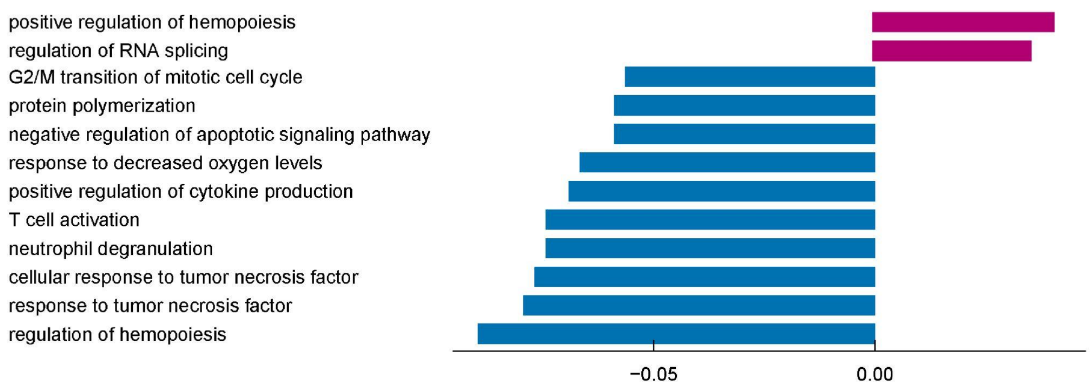

Figure. S11 Histogram showing differentially expressed genes' GO enrichment of Mast cells in AITD patients and controls. Violet bars represented the up-regulated pathways in patients, and blue bars represented the down-regulated ones.

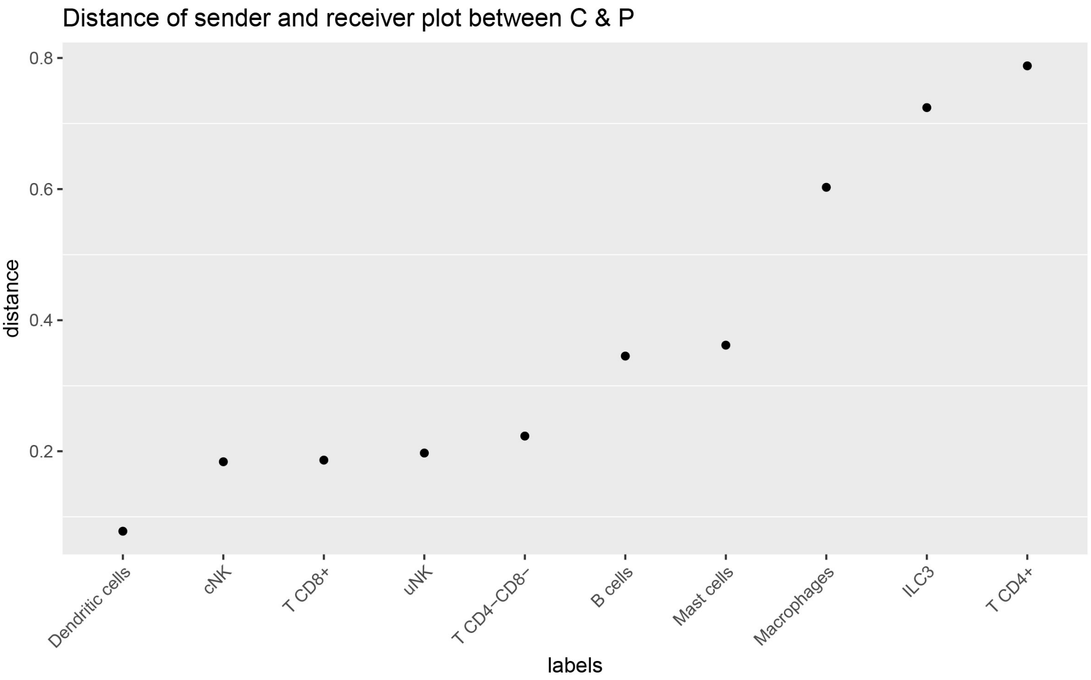

Figure. S12 Distance of jointly projecting and clustering signaling pathways of AITD patients and controls into a shared two-dimensional manifold according to their functional similarity.

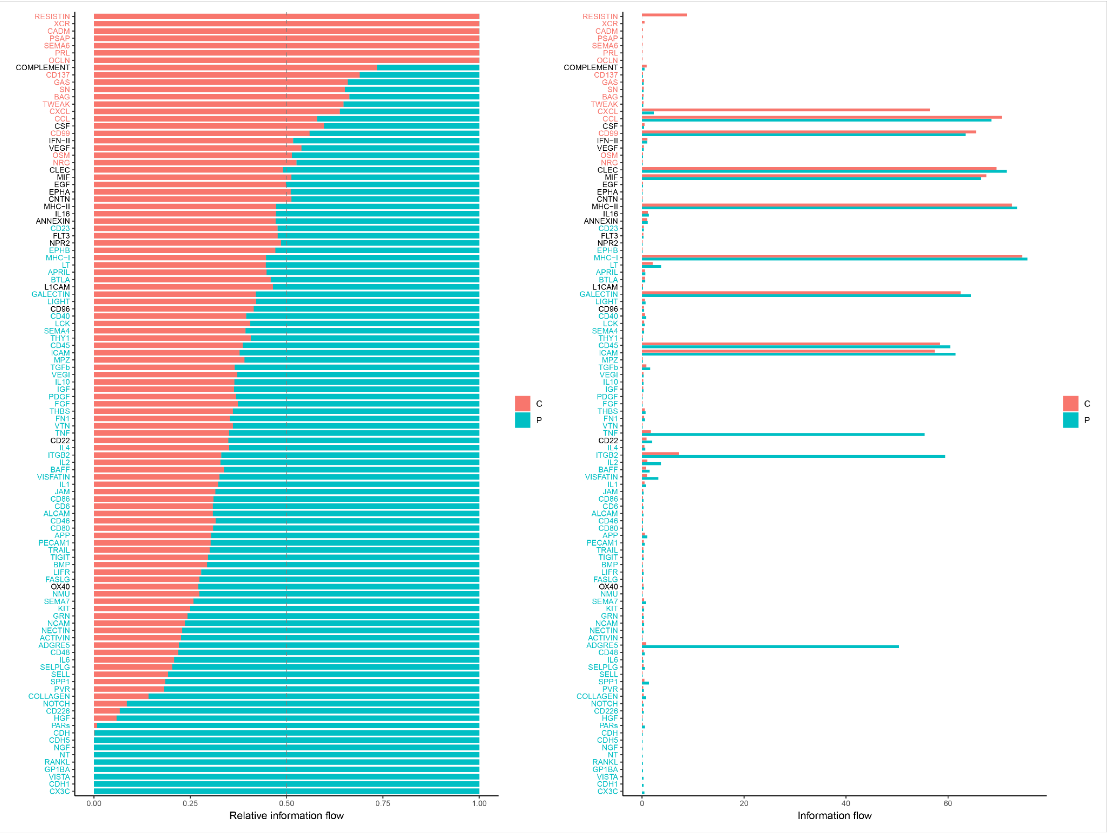

Figure. S13 All the detected signaling pathways were ranked based on their differences of overall information flow of scaled probability within the inferred networks between controls and patients, the up signaling pathways colored by red are more enriched in controls, and the down ones colored by green were more enriched in AITD patients.
